# Factors associated with long intensive care unit (ICU) admission among inpatients with and without diabetes in South Western Sydney public hospitals using the New South Wales admission patient data collection (2014–2017)

**DOI:** 10.1186/s12902-022-00933-8

**Published:** 2022-01-20

**Authors:** Uchechukwu L. Osuagwu, Matthew Xu, Milan K. Piya, Kingsley E. Agho, David Simmons

**Affiliations:** 1 Translational Health Research Institute (THRI), School of Medicine, Western Sydney University, Campbelltown, NSW 2560 Australia; 2 African Vision Research Institute (AVRI), University of KwaZulu-Natal, Durban, 4041 South Africa; 3 Diabetes, Obesity and Metabolism Translational Research Unit (DOMTRU), School of Medicine, Western Sydney University, Campbelltown, NSW 2560 Australia; 4 Macarthur Diabetes Service, Camden and Campbelltown Hospital, Campbelltown, NSW 2560 Australia; 5 School of Health Sciences, Western Sydney University, Campbelltown, NSW 2560 Australia

**Keywords:** Urban, Admissions, Hospitalisation, Intensive care, Primary admission diagnosis, Length of stay, Diabetes

## Abstract

**Background:**

Long stay in intensive care unit (ICU) is associated with poor outcomes, particularly in people with diabetes. It increases the financial burden of care and this is a challenge to the South Western Sydney region, which is already a hotspot for diabetes in Australia. This study compared ICU admission characteristics of people with and without diabetes and the factors associated with long ICU stay among patients admitted to public hospitals in this metropolitan health district from 2014 to 2017.

**Methods:**

Cross-sectional datasets on 187,660, including all ICU admissions in the New South Wales Admitted Patient Data Collection (APDC) from June 2014 – July 2017 in public hospital were extracted. Data on demographic and health insurance status, primary admission diagnosis using ICD-10, comorbidities including death among hospital inpatients aged ≥18 years residing in SWS were analysed. The ICU length of stay was the outcome variable and were classified into short stay (≤48 h) and long stay (> 48 h), and were examined against potential confounding factors using bivariate and multiple logistic regression analyses.

**Results:**

Our results showed higher ICU admissions in patients with diabetes than in those without diabetes (5% vs. 3.3%, *P* < 0.001) over three years. The median and interquartile range (IQR) of length of the ICU stay were similar in both groups [diabetes: 40 h, IQR = 16–88 h vs. non-diabetes: 43 h, IQR = 19–79 h]. The prevalence of long ICU stays among people with and without diabetes were 44.9% [95% CI 42.1, 47.7%] and 43.6% [95% CI 42.2, 44.9%], respectively. For both groups, increased odds of long ICU stay were associated with death and circulatory system disease admissions, while musculoskeletal disease admissions were associated with lower risk of long ICU stay. In the non-diabetes group, male sex, nervous system disease admissions and living in peri-urban areas were associated with higher odds of long ICU stay.

**Conclusions:**

The rate of ICU admissions among inpatients remain higher in people with diabetes. One in every two admissions to ICU had a long stay. Additional care for those admitted with circulatory system diseases are needed to reduce long ICU stay related deaths in SWS.

## Background

Diabetes is a major public health problem, with the worldwide prevalence of diabetes projected to double among adults from 463 million in 2019 to 592 million by 2035 [1]. Hospital inpatient care for people with diabetes makes up the largest medical expenditure in the US, accounting for 43% of the total medical costs in 2013 (US$409 billion) [2]. Admission to ICU could also significantly increase the medical cost, partly due to the high staffing ratios. The cost is significantly increased among those requiring longer time of intensive care treatment [3], which leads to increased burden for the hospital finances [4]. It is often accepted that the length of stay in the ICU averages 24 hours [5], and long ICU stay is associated with more complex procedures, serious complications and higher 1-year mortality [6].

In our previous work, we also found that, patients with diabetes had double the rate of ICU admissions of those without diabetes over three years [7]. A Critical Care Resources survey conducted in 2013/14 found that a total annual operational cost for ICU care in Australia was about $2119 million, which represents about 0.15% of gross domestic product (GDP) and 1.4% of total health care costs [8]. The cost of care for people with diabetes was about $210 per bed-hour or $5040 per ICU bed-day [9] but this is projected to increase by 436% by 2033 [10]. The increase in prevalence of diabetes coupled with the reported increase in diabetes related hospitalisations is likely to contribute to the increase in an already burdened health care system if proper measures to reduce admission and re-admission rates are not in place.

Residents of South Western Sydney (SWS) have poorer health outcomes and higher hospitalisation rates than the rest of the state [11]. The higher nurse to patient ratio in the ICU facilitates greater frequency of monitoring for example, the blood glucose measurement and the implementation of treatment protocols. This is in contrast to the lesser resources available on the wards for control of diabetes [12].

Limited data exist evaluating the influence of patient characteristics of people with and without diabetes on ICU admissions during a hospital stay, and previous studies are mainly from countries with different health systems [12–14]. Producing periodic prevalence estimates as well as identifying the predictors of ICU admission among inpatients with diabetes is needed to identify target groups for health promotion and diabetes management. This will also enable development of tools for scenario testing and effectiveness testing in future projections.

ICU length of stay (LoS) has also been suggested as a surrogate measure for assessing ICU resource utilization [13] as cost per day in ICU per patient is remarkably consistent across many diagnosis [14]. However, to date, there has been no Australian study of the effect of diabetes on ICU admissions. The paucity of such important data may lead to lack of uniform management policies across the country [15]. As part of a project to improve diabetes care in SWS local health district (LHD), the aim of this study was to investigate the factors associated with long ICU stay among people with and without diabetes admitted to SWS public hospitals across three financial years, between the 1st of July 2014 to 30th of June 2017.

## Methods

The New South Wales (NSW) APDC dataset [16] which was formerly known as the Inpatient Statistics Collection is administered by the NSW Health Department. The dataset is an annual collection and a census of all admitted patient services provided by NSW public and private hospitals. Public hospital data is reported weekly while private hospital data is reported monthly and detailed information regarding the APDC dataset is reported elsewhere [16].

The dataset was used to identify people from SWS and compare demographic and admission related data for all inpatient admissions provided by state hospitals using International Classification of Diseases 10th revision, Australian Modification (ICD-10-AM). People were coded by their place of residence rather than admission hospital [16]. Since acute hospital admissions in Australia mainly occur in public hospitals, for this study, only public hospital APDC data of episodes of care was used, which has previously been validated in people with diabetes, and other populations [17–20].

### Study population

Data used in this study were for people resident in SWS who were admitted to a public hospital over 3 years (July 1, 2014 to June 30, 2017). All admissions to the ICU were included. Data were available for residents from 6 out of 7 local government areas (LGAs) in SWS where a total of 187,660 adults aged ≥18 years with and without diabetes were admitted to public hospitals. Data were analysed to compare people with and without diabetes, and the effect of year of admission (2014–15, 2015–16 and 2016–17), residence (urban and peri-urban LGAs) based on the NSW Peri-urban Network of Council action plan [21], and ethnicity based on place of birth were assessed.

### Ethics approval

Approval for this study was obtained from the South Western Sydney Local Health District Human Ethics Research Committee as a quality improvement project (QA18/021), and study was conducted in accordance with the Declaration of Helsinki for human subject. Permission for use of de-identified data and waiver of individual consent was obtained from the data custodian. All methods were performed in accordance with the relevant guidelines and regulations.

### Statistical analysis

The outcome variable of this study was length of stay in ICU during admission, which was taken as binary: Short ICU stay defined as ICU stay of 1–48 h and Long ICU stay as > 48 h during admission, which was based from previous study [22]. Analyses were performed in STATA version 14.1 (Stata Corporation, College Station, TX, USA). Preliminary analyses involved frequency tabulations of all selected characteristics for people with and without diabetes who were admitted to intensive care unit during the study period. Death rate per 1000 person of people in ICU with and without diabetes was calculated for each group using a direct method. Prevalence estimates were examined against a set of all selected characteristics to assess the predictors of long ICU stay by diabetes status, including demographic, full hospital health insurance cover, primary admission diagnosis, and death. To plot long ICU stay by age in the category, we generated sampling weight to be ‘1’ and used the Survey command in Stata to estimate prevalence and their corresponding 95% confidence intervals (CI). Simple and multiple logistic regression were used to identify predictors of long ICU stay by diabetes status. In the unadjusted analyses, odds ratios (ORs) with 95% CI were calculated to assess the independent variable’s unadjusted risk.

A staged modelling technique was adopted for the multiple logistic regression analysis in which level-factors were entered progressively into the model to assess their relationship with the study outcome [23]. In the first stage, demographic factors were entered into the baseline multiple regression model to determine factors associated with the study outcome. Manual elimination process was used, and only variables associated with the outcome (*P* < 0.05) were retained as significant factors in the first model (model 1). In the second stage, financial factor (type of health insurance cover) was added to model 1, and those factors with *P* values < 0.05 were retained in the second model (model 2) after the elimination process was carried out. In the third stage, primary admission diagnosis (ICD-10) factors were added to model 2. As before, those factors with *P* values < 0.05 were retained in model three (model 3). Finally, the mode of separation from hospital (death) factor was added to model 3, and those factors with *P* values < 0.05 were retained in the final model. The ORs and their 95% CIs derived from the adjusted logistic regression models were used to determine the predictors of long ICU stay in NSW.

## Results

### Profile of the study group

Table 1 presents the characteristics of the participants admitted to ICU across SWS public hospitals between 2014 and 2017. Of the 187,660 inpatients from SWS, 6557 (3.5%) were admitted to the ICU over three years. People with diabetes were more likely to be admitted to ICU (*n* = 1216/24141, 5%) compared to those without diabetes (*n* = 5341/163519, 3.3%; *P* < 0.001). The median number of ICU hours dropped from 46 (25th–75th percentile interquartile range IQR 19–96) hours to 43 (IQR 19–85, *P* < 0.001) hours over the three years in the diabetes group but remained relatively stable in the non-diabetes group. The median length of ICU stay [40 h IQR 16–88 h versus 43 h IQR 19-79 h] was comparable between both groups. Of co-morbidities, there are more co-morbidities in people with diabetes than those without diabetes, and a few patients had primary endocrine diseases as a reason for admission.

**Table 1:** Characteristics of participants admitted to the ICU in South Western Sydney Local Health District hospitals (2014–2017)

Variables	Diabetes	Non-Diabetes
Year of admission	n (%)	n (%)
2014–2015	360 (29.6)	1665 (31.2)
2015–2016	393 (32.3)	1697 (31.8)
2016–2017	463 (38.1)	1979 (37.0)
Demography		
Age groups		
18-34 yrs	51 (4.2)	510 (10.0)
35-44 yrs	73 (6.0)	426 (8.0)
45-54 yrs	145 (11.9)	671 (12.6)
55-64 yrs	289 (23.8)	955 (17.9)
65-74 yrs	359 (30.0)	1202 (23.0)
75 yrs. and over	299 (24.6)	1577 (29.5)
Sex		
Women	476 (39.1)	2227 (41.7)
Men	740 (60.9)	3114 (58.3)
Marital status		
Married^*p*^	736 (60.8)	2962 (55.7)
Previously married^†^	315 (26.0)	1360 (25.6)
Never married	159 (13.1)	991 (18.7)
Country of birth		
Australia	501 (41.3)	2756 (51.7)
America	37 (3.1)	122 (2.3)
Asia	300 (24.7)	1028 (19.3)
Africa	26 (2.0)	102 (2.0)
Europe	242 (19.9)	994 (18.7)
Pacific	108 (8.9)	325 (6.1)
Residence^*‡*^		
Peri-urban	417 (34.4)	1835 (34.4)
Urban	799 (65.6)	3506 (65.6)
Hospital health insurance cover		
Full hospital cover	143 (11.8)	728 (13.6)
No hospital cover	1073 (88.2)	4613 (86.4)
Mode of separation from the hospital		
Deaths		
No	1081 (88.9)	4572 (85.6)
Yes	135 (11.1)	769 (14.4)
Primary admission diagnosis, ICD-10		
Diseases of the Nervous system	26 (2.1)	124 (2.3)
Diseases of the Respiratory system	126 (10.4)	721 (13.5)
Diseases of the Circulatory system	480 (39.5)	1596 (29.9)
Diseases of the Digestive system	106 (8.7)	603 (11.3)
Diseases of the Musculoskeletal/Connective system	22 (1.8)	89 (1.7)
Diseases of the Skin/Subcutaneous system	11 (0.9)	41 (0.8)
Diseases of the Endocrine/Nutritional/Metabolic system	114 (9.4)	79 (1.5)
Presence of any comorbidities		
No	331 (27.2)	2088 (39.1)
Yes	885 (72.8)	3253 (60.9)

*ICU* Intensive care unit, *ICD* International Classification of Diseases 10th revision (ICD-10), *p* includes de-facto, *†* Widowed/separated/divorced, *‡* Peri urban includes Wollondilly, Camden, Wingecarribee Local Government Areas (LGA) while Urban includes Campbelltown, Fairfield, Bankstown-Lidcombe LGAs

#### Age distribution of ICU participants

**Fig. 1:**
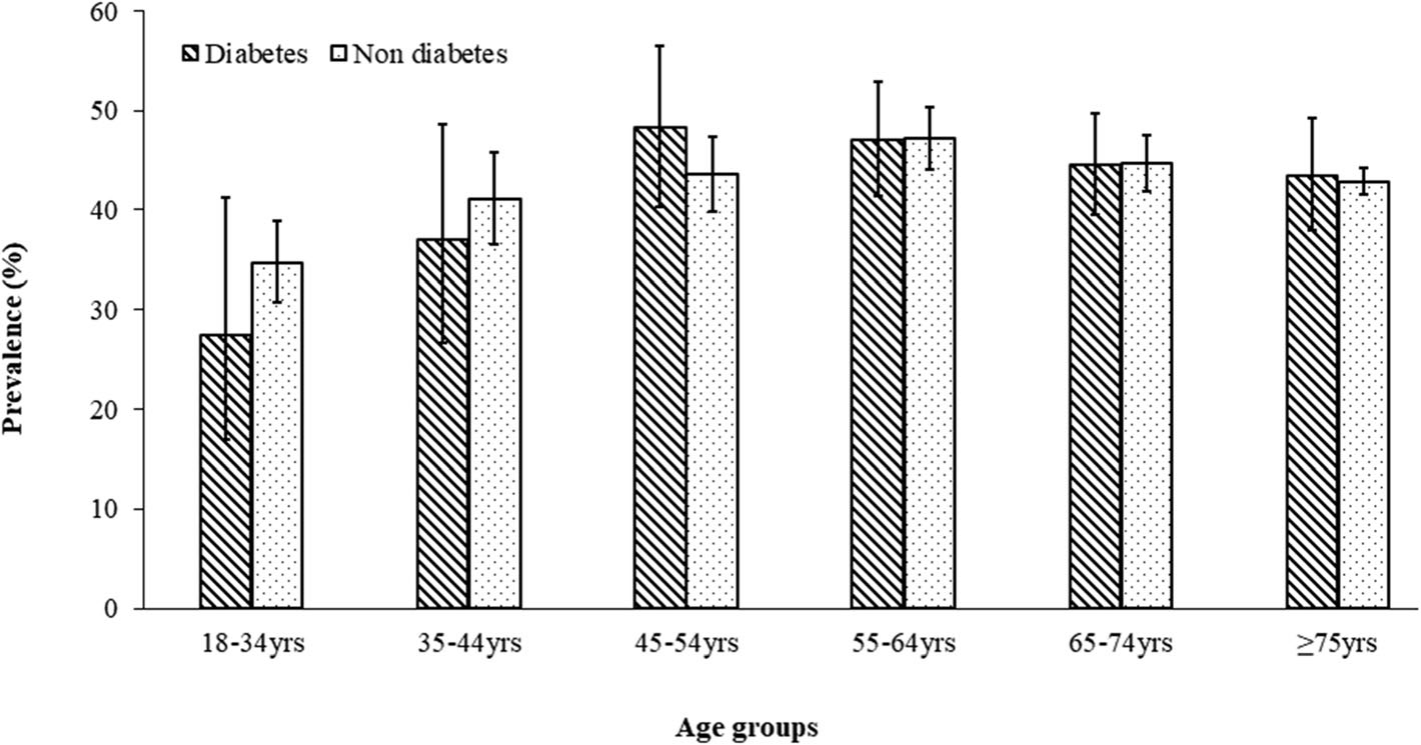
Prevalence of long intensive care unit (ICU) stay among admitted patients in South Western Sydney (SWS) public hospitals by age group. Error bars are 95% confidence intervals

Figure 1 shows the age specific prevalence of long ICU stay for those with and without diabetes. Whereas older people with diabetes (aged 45–54 years) spent more hours in ICU than those without diabetes, fewer hours in the ICU were recorded for the younger people with diabetes than their counterparts without diabetes.

#### Death in ICU

There was a lower death rate in ICU among those with diabetes (mortality rate of 111 per 1000 persons) compared with people without diabetes (143 per 1000 persons).

### Prevalence and unadjusted analysis of associated factors of long ICU stay

**Fig. 2:**
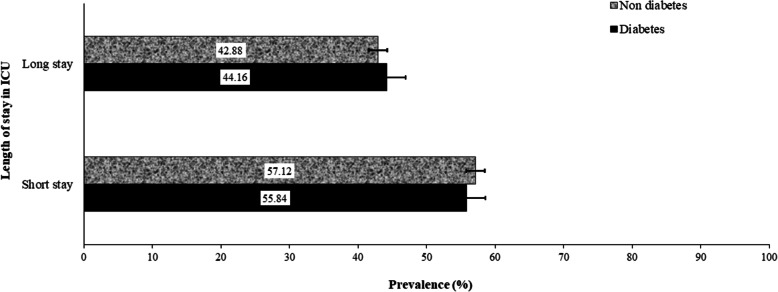
Prevalence of long and short intensive care unit (ICU) stay among admitted patients in South Western Sydney (SWS) public hospitals. Error bars are 95% confidence intervals

Figure 2 shows the prevalence of long stay (> 48 h) in ICU among people with and without diabetes. Approximately 45% of the adults admitted to ICU had a long ICU stay, including 547 with diabetes and 2326 without diabetes.

Table 2 presents the prevalence and unadjusted analysis of factors associated with long ICU stay including the primary admission diagnosis over the study years. The proportion of people with diabetes who reported a long ICU stay was significantly reduced by 9% in 2016–17 compared with 2014–15. Simple logistic regression analysis revealed that age > 44 years and having a circulatory system disease increases the odds for long ICU stay. The unadjusted analysis found that death during admission was also significantly associated with long ICU stay in people with diabetes.

**Table 2:** Prevalence and unadjusted odd ratios (OR) of factors associated with long ICU stay (> 48 h) among patients admitted to SWS hospitals by diabetes status (2014–2017). Bolded are significant associations

Variables	Diabetes			Non-Diabetes		
Year of admission	n (%)	OR	[95%CI]	n (%)	OR	[95%CI]
2014–2015	176 (48.9)	1.00		751 (45.1)	1.00	
2015–2016	174 (44.3)	0.83	[0.62, 1.11]	722 (42.5)	0.90	[0.79, 1.03]
2016–2017	187 (40.4)	**0.71**	**[0.54, 0.94]**	853 (43.0)	0.93	[0.82, 1.06]
Demography						
Age groups						
18-34 yrs	14 (27.5)	1.00		182 (36.0)	1.00	
35-44 yrs	27 (37.0)	1.55	[0.71, 3.37]	175 (41.0)	**1.31**	**[1.01, 1.71]**
45-54 yrs	70 (48.3)	**2.47**	**[1.23, 4.95]**	293 (44.0)	**1.45**	**[1.14, 1.84]**
55-64 yrs	136 (47.1)	**2.35**	**[1.22, 4.53]**	457 (47.9)	**1.68**	**[1.35, 2.10]**
65-74 yrs	160 (44.6)	**2.12**	**[1.11, 4.07]**	544 (45.3)	**1.52**	**[1.23, 1.88]**
75 yrs. and over	130 (43.5)	**2.03**	**[1.05, 3.92]**	675 (43.0)	**1.35**	**[1.09, 1.66]**
Sex						
Women	206 (43.3)	1.00		875 (39.0)	1.00	
Men	340 (45.9)	1.12	[0.89, 1.41]	1451 (46.6)	**1.35**	**[1.21, 1.51]**
Marital status						
Married^*p*^	319 (43.3)	1.00		1325 (45.0)	1.00	
Previously married†	151 (47.9)	1.03	[0.70, 1.51]	561 (41.2)	0.91	[0.77, 1.08]
Never married	73 (45.9)	0.85	[0.62, 1.16]	427 (43.0)	1.05	[0.91, 1.22]
Country of birth						
Australia	230 (45.9)	1.00		1186 (43.0)	1.00	
America	23 (62.2)	1.97	[0.99, 3.91]	50 (41.0)	0.91	[0.63, 1.32]
Asia	132 (44.0)	0.88	[0.66, 1.17]	475 (46.0)	1.14	[0.99, 1.32]
Africa	12 (46.1)	1.03	[0.47, 2.26]	48 (47.1)	1.12	[0.75, 1.66]
Europe	101 (41.7)	0.84	[0.62, 1.15]	424 (42.7)	0.99	[0.86, 1.15]
Pacific	47 (43.5)	0.89	[0.58, 1.35]	137 (42.2)	0.96	[0.76, 1.21]
Residence^*‡*^						
Peri-urban	201 (48.2)	1.00		855(46.6)	1.00	
Urban	345 (43.2)	0.82	[0.64, 1.03]	1471 (42.0)	**0.83**	**[0.74, 0.93]**
Hospital health insurance cover						
Full hospital cover	69 (48.2)	1.00		334 (45.9)	1.00	
No hospital cover	477 (44.4)	0.83	[0.54, 1.18]	1992 (43.2)	0.91	[0.78, 1.06]
Mode of separation from the hospital						
Deaths						
No	472 (43.7)	1.00		1913(42.0)	1.00	
Yes	74 (55.0)	**1.57**	**[1.09, 2.24]**	413 (53.7)	**1.56**	**[1.34, 1.82]**
Primary admission diagnosis, ICD-10 (Ref = No)						
Diseases of the Nervous system	15 (57.7)	1.75	[0.79, 3.83]	63 (51.0)	1.34	[0.94, 1.92]
Diseases of the Respiratory system	48 (38.1)	0.73	[0.50, 1.07]	311 (43.0)	0.96	[0.82, 1.12]
Diseases of the Circulatory system	246 (51.3)	**1.44**	**[1.14, 1.81]**	246 (51.0)	**1.44**	**[1.14, 1.81]**
Diseases of the Digestive system	51 (48.1)	1.19	[0.80, 1.77]	234 (38.8)	**0.83**	**[0.70, 0.98]**
Diseases of the Musculoskeletal/Connective system	4 (18.2)	**0.28**	**[0.09, 0.82]**	24 (27.0)	**0.49**	**[0.30, 0.78]**
Diseases of the Skin/Subcutaneous system	5 (45.4)	1.05	[0.32, 3.47]	9 (21.9)	**0.37**	**[0.18, 0.78]**
Diseases of the Endocrine/nutritional/metabolic system	546 (44.9)	**0.58**	**[0.34, 0.87]**	31 (39.0)	0.81	[0.51, 1.29]
Presence of any comorbidities						
No	140 (42.3)	1.00		806 (38.6)	1.00	
Yes	397 (44.9)	1.11	[0.86, 1.43]	1484 (45.6)	**1.33**	**[1.19, 1.49]**

*ICU* Intensive care unit, *ICD* International Classification of Diseases 10th revision (ICD-10), *n* number, *OR* odd ratio, *p* includes de facto, † Widowed/separated/divorced, ‡ Peri urban includes Wollondilly, Camden, Wingecarribee Local Government Areas (LGA) while Urban includes Campbelltown, Fairfield, Bankstown-Lidcombe LGAs

In the non-diabetes group, men, and those aged 35 years and above, people whose primary diagnosis during admission was a circulatory system disease and those with any comorbidity during admission were more likely to report a long ICU stay. In addition, people without diabetes admitted to the ICU for greater than 48 h were more likely to die than survive during that admission (OR, 1.56, 95% CI: 1.34, 1.82).

### Factors associated with long ICU stay among admitted people with and without diabetes

Table 3 shows the adjusted odd ratios for the associations with long ICU stay in both groups. After adjusting for the potential confounders, people with diabetes admitted to the hospital in 2016–17 and those with a disease affecting the musculoskeletal system were less likely to have a long ICU stay than those admitted in 2014–2015 and did not have a disease affecting the musculoskeletal system. The presence of a circulatory system disease during admission increased the likelihood of long ICU stay by 1.4 folds.

**Table 3:** Adjusted odds ratio (aOR) of factors associated with long ICU stay among admitted adults in South Western Sydney Public hospitals. *Bolded are significant predictors*

Variables	aOR	[95% CI]
Diabetes		
Year of admission		
2014–2015	1.00	
2015–2016	0.83	[0.62, 1.10]
2016–2017	**0.72**	**[0.54, 0.95]**
Primary admission diagnosis, ICD-10 (Ref = No)		
Diseases of the circulatory system	**1.40**	**[1.11, 1.77]**
Diseases of the musculoskeletal system	**0.31**	**[0.10, 0.94]**
Death	**1.59**	**[1.10, 2.28]**
Non-diabetes		
Year of admission		
2014–2015	1.00	
2015–2016	0.90	[0.78, 1.04]
2016–2017	0.95	[0.83, 1.09]
Sex		
Women	1.00	
Men	**1.27**	**[1.14, 1.42]**
Residence^*‡*^		
Peri-urban	1.00	
Urban	**0.83**	**[0.74, 0.93]**
Primary admission diagnosis, ICD-10 (Ref = No)		
Diseases of the nervous system	**1.51**	**[1.05, 2.16]**
Diseases of the circulatory system	**1.60**	**[1.42, 1.80]**
Diseases of the musculoskeletal system	**0.58**	**[0.36, 0.93]**
Death	**1.57**	**[1.34, 1.83]**

*ICU* Intensive care unit, *aOR* adjusted odd ratio, ‡ Peri urban includes Wollondilly, Camden, Wingecarribee Local Government Areas (LGA) while Urban includes Campbelltown, Fairfield, Bankstown-Lidcombe LGAs

After adjusting for all the potential confounders, men, people without diabetes who had a disease affecting the nervous and circulatory systems were more likely to have a long ICU stay, where as those without diabetes who were diagnosed with a musculoskeletal system disease on admission had reduced odds for long ICU stay. The urban residency was associated with lower odds for long ICU stay compared with rural residency [aOR 0.83, 95% CI 0.74, 0.93], and in both diabetes and non-diabetes group, patients with a long ICU stay were more likely to die than survive during admission compared to those with shorter ICU stay.

## Discussion

This study investigated the prevalence and factors associated with long ICU stay using admission data for SWS residents over three years. The study found that patients with diabetes who are admitted to SWS hospitals have a greater likelihood of being admitted to the ICU compared with those without diabetes. The median length of ICU stay was comparable between both groups, but those admitted to ICU for > 48 h have poorer outcomes and increased risk of death. Cardiovascular disease was associated with long stay in ICU in patients with and without diabetes, while nervous system disease and male sex are additional associated factors for those without diabetes.

Past studies on ICU are mainly from countries with different health systems [12–14]. While other investigators have reported findings similar to ours [13, 24], the present study provided a detailed assessment of the factors associated with long ICU stay in an Australian setting using a nationally representative database. The findings that long ICU stay was associated with increased risk of death in admission is contradictory to previous report of that the outcomes in people with long and short ICU stay in Saudi Arabia were similar [13]. Our findings must be interpreted in the proper context, keeping in mind that the definition of long ICU stay in the previous study was > 14 days, involved only those in the medical/surgical ICU of a tertiary-care teaching hospital [13]. In another study [22] where prolonged stay was defined as > 48 h in ICU, the authors found that 19% of patients who underwent coronary bypass surgery had a prolonged ICU stay and this was a risk factor for death and readmission. Although some studies have also used a different definition for prolonged ICU stay (range from 3 to 14 days), they included only surgical patients [25, 26], people with diabetes complications [27] or those with intracerebral haemorrhage [28]. The study conclusions were limited to a very small number of patients (< 5% of the original population), but all of the studies agree that prolonged ICU stay was associated with higher mortality rate (range from 15 to 33%). Nevertheless, half of the patients in the present study were in ICU for > 48 h and the present findings suggest that this high-risk group is worth investing to reduce the cost of care. This information is useful as decisions about continuing or withholding aggressive intensive care management based on long ICU stay may yield better outcomes. Emphasis should also improve ICU efficiency for those with and without diabetes without compromising the level of care.

The study identified certain factors associated with long ICU stay among admitted people in SWS, which can help plan strategies to improve resource utilization. The presence of a circulatory system or nervous disease in people with and without diabetes increased the likelihood of long ICU stay. Since patients with comorbidities stay longer and utilize more resources [13, 29], they constitute groups of patients worth investing in. It is also important that nurses recruited for ICU care have the proper background to care for patients with circulatory and nervous system diseases. It appears that patients admitted in the last year of this study (2016–2017) were less likely to stay longer than 48 h in ICU compared with those admitted in 2014–15 indicating some improvement in ICU care across SWS public hospitals. Another study showed a similar decrease in severity of disease on ICU admission, after 8 years, more so following the addition of additional medical ICU beds [30].

The increased likelihood for ICU admission in people with diabetes compared with those without diabetes in Australia may reflect pre-existing comorbidity in people with diabetes. Among critically ill patients in India, 13.9% of the 1283 admissions in ICU over 4 years were established cases of diabetes, with 5.0% diagnosed as diabetes after admission. Past studies [17, 31] have also reported significant interaction between pre-existing hyperglycaemia and the association between acute glycaemia and mortality among people with diabetes in ICU [17]. In-hospital control of glucose in people with diabetes receiving ICU care is associated with a reduced length of hospital stay and lower mortality rate [31]. These findings suggest that it is even more important to deal with diabetes management in primary care, help prevent hospital admission in the first place, and reduce the risk of ICU admissions and mortality.

The lower ICU admission rate seen in peri urban regions of SWS may be related to the suggestion that the sickest patients in these regions may die before admission to an ICU because of longer transfer times or through a lack of timely access to appropriate critical care services [32–34]. It is also possible that hospitals in the peri-urban regions are under less pressure to admit patients who are unlikely to survive to ICUs [33]. The cost of hospital admission to patients may be a factor in the reduced risk of admissions in other countries, but in Australia, public hospitals do not charge for ICU care and hospital stay for Australian residents/citizens. Therefore, in this study, having private health insurance coverage does not appear to influence the length of stay in ICU. In a study conducted in France among a low-income population with free access to health care, the researchers found higher hospitalisation and mortality rates for many diseases among people with access to free government health care than those without free access [35]. However, ICU care was not assessed.

ICUs admission is associated with greater utilization of hospital resources [13] and more in people with diabetes [36] with studies suggesting that this may be due the interaction between the longer LoS in hospital and the potential to receive emergency or intensive care during admission [36–38]. Lee et al [39] suggested that it cost the Australian government an average of A$5764 per admission to care for an individual with diabetes, and the cost increases by 1.3 times in people with diabetes who have both micro-and macrovascular complications. In this study, we found that people with diabetes needed more ICU care indicating the presence of complications. In a study using the Hospital Pricing Authority, researchers found that the Australian government spent about $210 per bed-hour or $5040 per bed-day for any individual who is admitted to ICU [8]. The estimated cost are likely to be higher in people with diabetes, because calculations were based on 2013 cost data, they did not consider the non-healthcare cost including costs of adverse events, glucose monitoring [39] and/or the impact of undiagnosed diabetes [36] both of which attract a higher cost. The group of patients with long-stay should be targeted for promotion of more optimal bed utilization by decreasing ICU length of stay. In the UK, the median cost per patient day in an ICU was estimated at US$1356 (range $1242–1745) [40]. A previous systematic review suggested that a regular source of primary care and a well-controlled HbA1c would reduce the likelihood of hospitalisation in people with diabetes [41].

Strengths of this study include the provision of evidence of increased ICU care for general admissions of people with diabetes in Australia, and the large number of people included in the study as well as the use of place of residence rather than admitting hospital to define the population. The latter removes the bias associated with secondary/tertiary care centres, as well as including people admitted to hospitals outside the district. The main limitations of the study are due to the nature of the data being used (secondary data analysis). The coding of diabetes relies on accurate discharge summaries and it is very likely that diabetes is under-reported due to coding errors as with all administrative databases [42]. Although a previous study [43] found good agreement between self-reported diabetes and coded diabetes, a much higher inpatient prevalence of diabetes was found in an audit of inpatients in Melbourne [44]. Due to the higher length of stay for diabetes, the point prevalence will always be higher in hospital, but not as much when datasets like APDC look at all admissions. Data on the type of diabetes was not extracted, country of birth was used as ethnicity and indigeneity are not routinely available. Additionally, the use of de-identified data did not allow for the identification of multiple admissions, and data were not available for one of the seven LGAs in the district. No body mass index or glucose data was used in this study as these were not captured in the database, and socioeconomic status data were also not available. Finally, duration of diabetes and glycaemic control were not available, and thus could not be adjusted for.

## Conclusions

This study used data from NSW APDC to demonstrate that about one-half of the adult inpatients in SWS admitted to ICU in public hospitals over three years stayed in ICU for more than 48 h during same admission. The rate of ICU admissions among inpatients remain higher in people with diabetes. Admissions due to circulatory system diseases was a risk factor for long ICU stay in both groups while nervous system disease and male gender were risk factors for long ICU stay among those without diabetes. Some of the risk factors identified in this study are likely to reflect the high local diabetes prevalence associated with lower socioeconomic groups and ethnic minority populations. New strategies that target the groups at the highest risk identified here should be considered. Such an approach could bridge the gap between Australian State and Federally funded services to reduce both hospitalization and ICU admissions in order to improve outcomes in people with diabetes.

## Data Availability

All data generated or analysed during this study are included in this published article. Data is also publicly available on request from the Centre for Health Record Linkage, which is managed by the NSW Ministry of Health. Available at: https://www.cherel.org.au/data-dictionaries#section1

## Abbreviations

SWS: South Western Sydney
NSW: New South Wales
SWSLHD: South Western Sydney Local Health District
LGA: Local Government Area
ABS: Australian Bureau of Statistics
NSW APDC: New South Wales Admitted Patient Data Collection
ICU: Intensive Care Unit
HbA1c: Glycated haemoglobin A1c
CVD: Cardiovascular disease
LoS: Length of stay
OR: Odds ratio
aOR: Adjusted odds ratio
ICD-10: International classification of diseases, tenth version
CI: Confidence interval
